# Multi‐objective Bayesian algorithm automatically discovers low‐cost high‐growth serum‐free media for cellular agriculture application

**DOI:** 10.1002/elsc.202300005

**Published:** 2023-06-28

**Authors:** Zachary Cosenza, David E. Block, Keith Baar, Xingyu Chen

**Affiliations:** ^1^ Department of Chemical Engineering University of California Davis USA; ^2^ Department of Viticulture and Enology University of California Davis USA; ^3^ Department of Neurobiology, Physiology, and Behavior and Physiology and Membrane Biology University of California Davis USA

**Keywords:** Bayesian optimization, cellular agriculture, design of experiments, multi information source optimization

## Abstract

In this work, we applied a multi‐information source modeling technique to solve a multi‐objective Bayesian optimization problem involving the simultaneous minimization of cost and maximization of growth for serum‐free C2C12 cells using a hyper‐volume improvement acquisition function. In sequential batches of custom media experiments designed using our Bayesian criteria, collected using multiple assays targeting different cellular growth dynamics, the algorithm learned to identify the trade‐off relationship between long‐term growth and cost. We were able to identify several media with >100% more growth of C2C12 cells than the control, as well as a medium with 23% more growth at only 62.5% of the cost of the control. These algorithmically generated media also maintained growth far past the study period, indicating the modeling approach approximates the cell growth well from an extremely limited data set.

**Abbreviations**
E8/B8: Essential 8
MOO: multi‐objective optimization
MOBO: multi‐objective Bayesian optimziation
DMEM: Dulbecco's Modified Eagle Medium
FBS: fetal bovine serum
BO: Bayesian optimization
GP: Gaussian process
L‐BFGS‐B: limited memory bounded Broyden Fletcher Goldfarb Shanno algorithm

## 1 INTRODUCTION

In this work we applied an active learning approach to design serum‐free media, which is a necessary precondition to the development of cellular agriculture. In cellular agriculture, animal products are grown in bio‐reactors [1] in an attempt to be more resource efficient and ethical than traditional animal agriculture. Because of the enormous potential cost of media [2], the need to not use animal‐products (like serum) and the need to grow larger amounts of cells than in traditional biotechnology, we set out to design a medium that is inexpensive, serum‐free, and supports long‐term proliferation of as many animal cells as possible. The work by [3] on Essential 8 (E8 or B8) media is a good framework to establish serum‐free media. They developed E8/B8 for human induced pluripotent stem cell proliferation and stability based on the combination of the Dulbecco's Modified Eagle Medium (DMEM)/F12 basal medium and supplementation with insulin, transferrin, FGF2, TGFβ1, ascorbic acid, and sodium selenite [4] took this approach and, by screening multiple growth factors and hormones using a one‐factor‐at‐a‐time approach, developed an albumin‐enriched B8 formula for the proliferation of bovine satellite cells. A shortcoming of this approach, which is acknowledged, was that they only looked at a very limited set of variables that may affect cell growth. One way of sifting through this large experimental design space is active learning, where data is collected to maximally inform the next decision. This process repeats itself until the user desires to stop. Previous work by [5] (bacteria culture) and [6, 7] (C2C12 cell culture) demonstrate that active learning techniques are very resource‐efficient in optimizing experimental systems with many variables. In this manner an active learning algorithm mimics how real scientists observe data and alter future experiments based on updated expectations.

The serum‐free medium itself must contain vitamins, trace elements, carbohydrates, amino acids, and salts, with additional proteins that replace serum [8]. These serum‐replacing components are particularly expensive and militate for a multi‐objective optimization (MOO) approach to optimizing cell culture media in order to explore the trade‐off between cost and cell growth. In MOO problems, there is often no single point that dominates the entire design space, so becomes a matter of finding sets of points that fall on the trade‐off curve. Cell culture media design, particularly for cellular agriculture, is inherently a MOO problem because improved growth is often found with expensive components [9] used central composite designs to evaluate the effect of several components on a desirability function parameterization of lipid content, carbohydrate consumption and biomass accumulation. In work done to optimize cytokine dosing [10], trained a regularized polynomial model and used a derivative‐free optimizer to find the conditions that maximized a desirability function of cell populations. In work by [11], genetic algorithms vector evaluated genetic algorithm (VEGA) and strength Pareto evolutionary algorithm (SPEA) were used to maximize chemical conversion while maintaining biomass of the cyanobacteria organism [12] used a genetic algorithm MOGA to maximize plant culture biomass and minimize system cost.

Here, we meld MOO with active learning using Bayesian optimization (BO) in a multi‐objective Bayesian optimization (MOBO) approach to design media experiments. Specifically, we utilized the noisy expected hypervolume improvement function described in [13] to rank sets of experimental conditions that could be done in batches in a wet lab. We have previously used a multi‐information source Gaussian process model described in [14] to successfully optimize cell culture media with multiple assays to robustly describe long‐term cell proliferation [7]. We will extend this model again to model long‐term cell growth in our serum‐free system and use the models predictions to solve the MOBO problem. In Section 2 we will discuss the laboratory materials needed to solve our media design problem, including the cells and chemicals needed, as well as the mathematical derivation of the acquisition function used to solve the MOBO problem. Then in Section 3 the results will be presented, followed by Section 4 with the discussion of the implications of the results.

## 2 MATERIALS AND METHODS

### 2.1 Serum‐free cells

Recent work by [15] shows that merely seeding cells in serum‐fre e media without additional preparation is insufficient in optimizing serum‐free media, as the cells do not have time to adjust to the environment. A more robust approach is to slowly adapt a cell line to serum‐free conditions over multiple passages [16]. Sometimes this requires attachment factors or extra‐cellular matrix (ECM) material to allow adherent cells to affix themselves to the surface of the culture dish. For a fully animal component‐free medium, ECM substitutes like Matrigel may be replaced by dilution cloning. To get the C2C12 cells American Type Culture Collection (ATCC) to proliferate in serum‐free conditions, they were first adapted to survive in Essential 8 (Gibco) (E8) medium by passaging the cells, starting in DMEM (Gibco) and 10% fetal bovine serum (FBS) (BioWest), in increasing amounts of E8. Once E8 comprised >90% v/v of the medium, cell growth slowed and Matrigel (Corning) was needed to provide ECM. With the Matrigel, the new C2C12 line survived fully in E8. Next we used dilution cloning to select a subset cell line from these cells that could survive without Matrigel. This was done by seeding 1 cell/well of Matrigel‐conditioned cells in 24 well plates (Cellstar, Greiner Bio‐One) without Matrigel and isolating the single cell that survived and managed to proliferate. The surviving cell population was frozen in Synth‐a‐Freeze (Gibco) at their fourth passage in ‐196°C liquid N_2_ and are the cells used in the remainder of this work.

### 2.2 Media components

The media design space was based on the E8/B8 formulation [3] comprised of basal medium, FGF2, TGFβ1, insulin, transferrin, ascorbic acid, and sodium selenite. We chose to supplement this with nine growth factors which have either been found to improve cell proliferation in [7] or by expert opinion. Because the basal component is comprised of >30 individual components it was broken down into groups based on function in cell culture. These component groups (essential and non‐essential amino acids, vitamins, salts, trace metals, DNA precursors, fatty acids) were varied during the optimization campaign by the algorithm which we discuss in later sections. Components believed to have significant effects on growth (carbohydrates, ascorbic acid, sodium selenite) were individually varied as well. NaCl was separated from the general salts group because it has a large effect osmolarity. All components and groups of components are shown in detail in Table 1 and Appendix 1.

**TABLE 1:** Serum‐Free Medium Design Space|all components were stored as per manufacturers instructions in stock solutions described in Appendix 1. The concentration (mg/mL) of all media was between the minimum and maximum listed. The cost shown is a unitless coefficient that corresponds to the marginal USD cost on the [0,1] scale. Sterile cell culture grade water was used to make up the remaining volume not taken up by the components.

**Abrev**.	**Component**	**Conc. Min**	**Conc. Max**	**Cost**
NEAA	^a^Non‐Essential Amino Acids	0.5x	5x	0
EAA	^a^Essential Amino Acids	0.5x	5x	0
V	^a^Vitamins	0.5x	5x	0
Salt	^a^Salts	0.5x	5x	0
Metal	^a^Trace Metal	0.5x	5x	0
DNA	^a^DNA Precursor	0.5x	5x	0
Fat	^a^Fatty Acid	0.5x	5x	0
SS	Sodium Selenite	7.00E‐06	7.00E‐05	0
AA	Ascorbic Acid	0.03	0.30	0
Gluc	Glucose	1.35	13.50	0
Gluta	Glutamine	0.22	2.20	0
Pyruv	Sodium Pyruvate	0.03	0.30	0
NaCl	Sodium Chloride	1.40	14.0	0
I	Insulin	0.01	0.10	0.03
T	Transferrin	5.00E‐03	0.05	0.004
FGF2	FGF2	3.00E‐05	3.00E‐04	0.63
TGFb1	TGFβ1	1.00E‐06	1.00E‐05	0.09
EGF	EGF	0	2.50E‐05	0.003
P	Progesterone	0	2.50E‐05	0
Estra	Estradiol	0	1.25E‐05	0
IL‐6	IL‐6	0	6.25E‐05	0.08
LIF	LIF	0	1.25E‐05	0.02
TGFb3	TGFβ3	0	1.60E‐05	0.04
HGF	HGF	0	2.50E‐05	0.03
PDGF	PDGF	0	2.50E‐05	0.03
PEDF	PEDF	0	2.50E‐05	0.04

^a^ The upper and lower bound concentration for these grouped variables was set relative to stock concentrations in the Appendix 1. All media have a sodium bicarbonate concentration of 2.44 mg/mL and were stored at 5°C for no longer than 8 days.

### 2.3 Cell growth experiments and assays

We utilized a multi‐information source Bayesian model to combine “cheap” measures of cell biomass with more “expensive” but higher quality measurements in order to predict long‐term medium performance. We refer to the simpler and cheap assays as “low‐fidelity”, and more complex and expensive assays as “high‐fidelity”. In this work, low‐fidelity experiments were conducted with AlamarBlue and LIVE stain, common indirect chemical indicators of cell proliferation. High‐fidelity experiments were conducted by cell counting with trypan blue. To start a set of experiment, vials of adapted C2C12 cells were thawed to 25°C and the freezing medium was removed by centrifugation at 1500 × g for 4 min. The centrifuged cell pellet was resuspended in 17 mL of E8 (Gibco) and placed on 15 cm sterile plastic tissue culture dishes (Cellstar, Greiner Bio‐One). Cells were incubated at 37°C and 5% CO_2_ for 48 h. Cells were harvested using tripLE solution (Gibco), diluted in PBS, and counted using a Countess II with trypan blue exclusion and disposable slides (Invitrogen). With the known concentration of cells, 96 well plates (for the low‐fidelity IS) were seeded at 2000 cells/well (25 μL of PBS/cell inoculum and 75 μL of test medium) and 6 well plates (for the high‐fidelity IS) were seeded at 60,000 cells/well (750 μL of PBS/cell inoculum and 2250 μL of test medium). The final density of both formats was 20,000 cells / mL of PBS and medium.

After 72 h, all wells were measured using the different fidelity methods. AlamarBlue (Invitrogen) and LIVE stain (Biotium) assay required addition of a chemical indicator into the 96 well plates followed by collection of absorbance or fluorescence signals (this was done using Molecular Devices, ImageXpress Pico) according to manufacturer instructions. A Passage 1 cell count was also done in the 6 well plate format using the automatic cell counter. However, these three ways of measuring growth may not represent the long‐term proliferation required in cellular agriculture because (1) they were only exposed to the media for 72 h and (2) were not exposed to an additional passage in their custom media which may affect growth. Therefore, a high‐fidelity experiment is conducted where the Passage 1 cells are re‐seeded for an additional 72 h and counted again to create a fourth and final Passage 2 metric of proliferation. This additional 72 h period is why it is considered a long‐term cell growth metrics, but also why it is more tedious to use to optimize a complex media. We now move on to how to model the high‐fidelity growth data using low‐fidelity interactions as well as how to rank the quality of a medium.

### 2.4 Basics of BO

In standard BO, a function *g* is modeled using a Gaussian Process (GP) [14], characterized by a mean μ_0_ and covariance Σ where $g\left(x\right)∼N\left(\mu_{0},\Sigma \right)$. This “prior” influences the function through the covariance Σ and prior mean μ_0_, which models the relationship between any two points *x* and $x^{\prime }$. We have chosen the squared exponential kernel because it performed well in our previous work [7].

(1) $$\Sigma \left(x,x^{\prime }\right)=\sigma_{f}^{2}exp\left(-1/2\sum_{k=1}^{p}\frac{{\left(x_{k}-x_{k}^{\prime }\right)}^{2}}{\lambda_{k}^{2}}\right)$$

If we collect *N* data points of inputs $X_{N}=\left[x_{1}\text{…}x_{N}\right]$ and outputs $Y_{N}=\left[y_{1}\text{…}y_{N}\right]$ from the process y(x)=g(x)+ε we get the posterior distribution $g\left(x\right)|X_{N},Y_{N}∼N\left(\mu \left(X_{N}\right),\Sigma \left(X_{N},X_{N}\right)\right)$ where the mean and variance of g(x) are given by Equations (2) and (3) respectively for homoscedastic noise $\Sigma_{\varepsilon }=\sigma_{\varepsilon }^{2}\times I$ with process noise variance $\sigma_{\varepsilon }^{2}$. Notice that this prior is parameterized by hyper‐parameters μ_0_, $\lambda_{k}$, $\sigma_{f}$, and $\sigma_{\varepsilon }$. These are “learned” from the data by maximizing the log‐likelihood function (which will not be discussed here for brevity).

(2) $$\mu \left(x\right)=\mu_{0}+\Sigma \left(X_{N},x\right){\left(\Sigma \left(X_{N},X_{N}\right)\right)}^{-1}\left(Y_{N}-\mu_{0}\right)$$

(3) $$\sigma^{2}\left(x\right)=\Sigma \left(x,x\right)-\Sigma \left(X_{N},x\right){\left(\Sigma \left(X_{N},X_{N}\right)\right)}^{-1}\Sigma {\left(X_{N},x\right)}^{T}$$

We introduce fidelity information by modifying Equation (1) with an indicator function $1_{m\neq 0}1_{m=m^{\prime }}$, where m=0 indicates an experiment is high‐fidelity. Now, an experiment not only contains media concentrations *x*, but a fidelity code *m*. By applying Equations (4) to (2) and (3), we can make predictions using concentration *x* and fidelity *m*. We use the same kernel architecture as Equation (1) to model low‐fidelity $\Sigma_{m}\left(x,x^{\prime }\right)$ and high‐fidelity covariance $\Sigma_{0}\left(x,x^{\prime }\right)$.

(4) $$\Sigma \left(x_{m},x_{m^{\prime }}^{\prime }\right)=\Sigma_{0}\left(x,x^{\prime }\right)+1_{m\neq 0}1_{m=m^{\prime }}\Sigma_{m}\left(x,x^{\prime }\right)$$

### 2.5 MOBO acquisition function

Turning now to how to rank a set of experiments, we have chosen hypervolume metric HV(x) to rank the quality of *p* media combinations based on S=2 criteria, predicted growth μ(x) and cost c(x). To compute cost use $c\left(x\right)=c_{min}+\Sigma_{j=1}^{p}c_{j}x_{j}$ where $c_{j}$ is a scaled marginal cost of each media component whose coefficients can be found in Table 1. If the *s*th output (to maximize) is $f_{s}\left(x\right)$ (use Equation (2) to solve this at m=0 if *s* references growth and −c(x) if *s* references cost) relative to a minimum reference point $l_{s}$ then HV(x) is the product of $f_{s}\left(x\right)-l_{s}$ for each output [17]. The “+” operator in Equation (5) sets HV(x)=0 if $f_{s}\left(x\right)-l_{s}\leq 0$ (this acts as a threshold).

(5) $$HV\left(x\right)=\prod_{s=1}^{S}{\left[f_{s}\left(x\right)-l_{s}\right]}^{+}$$

To compute the “improvement” in Equation (5) we reformulate the above expression into the product of the minimum between a max‐value called $u_{s}$, and $f_{s}\left(x\right)$ [13] where $z_{s}\left(x\right)=min\left\{u_{s},f_{s}\left(x\right)\right\}$. As discussed in the cited Daulton papers, a box decomposition algorithm can be used to quickly compute HVI(x) by breaking down the above computation into a piece‐wise integration across *K* rectangles defined by upper and lower vertices $u_{s}$ and $l_{s}$. We numerically integrate over the rectangles to get the approximation of the hyper‐volume improvement function HVI(x).

$$HVI\left(x\right)=\prod_{s=1}^{S}{\left[z_{s}\left(x\right)-l_{s}\right]}^{+}$$

$$HVI\left(x\right)\approx \sum_{k=1}^{K}\prod_{s=1}^{S}{\left[z_{s,k}\left(x\right)-l_{s,k}\right]}^{+}$$

Because we can run multiple experiments in a single batch, we can again reformulate HVI(x) into the “multi‐point” qHVI(X) where we wish to predict the best *q* set of experiments *X*. This can be done using the *inclusion‐exclusion* principle for overlapping sets. In practice, this means summing across *q* points $\sum_{j=1}^{q}{\left(-1\right)}^{j+1}$ and modifying the improvement calculation to incorporate all subsets of the proposed candidate pool *X* of size *j* for j=1⋯q. This additional calculation prevents double counting of any *q* overlapping hyper‐volume sets. Note that $z_{s,k,X_{j}}=min\left\{u_{k},f_{s}\left(X_{i,1}\right)\cdots f_{s}\left(X_{i,j}\right)\right\}$. Finally, because we have a statistical model of the *S* outputs (technically c(x) is deterministic), we formulate an “expected” multi‐point improvement as the integral over the posterior distribution over the previous formulation, or $qEHVI\left(X\right)=\frac{1}{N}\sum_{t=1}^{N}qHVI\left(X\right)$ in the case of monte‐carlo (MC) sampling of *N* points (MC is needed because there is no analytical solution to qHVI(X)).

$$qHVI\left(X\right)=\sum_{X_{j}\in \Omega }\sum_{j=1}^{q}\sum_{k=1}^{K}\prod_{s=1}^{S}{\left(-1\right)}^{j+1}{\left[z_{s,k,X_{j}}\left(x\right)-l_{s,k}\right]}^{+}$$

$$\begin{array}{ccc} qEHVI(X) & = & \frac{1}{N}\sum_{t=1}^{N}\sum_{X_{j}\in \Omega }\sum_{j=1}^{q}\sum_{k=1}^{K}\prod_{s=1}^{S}{\left(-1\right)}^{j+1}\\ & & \times \;{\left[z_{s,k,X_{j},t}\left(x\right)-l_{s,k}\right]}^{+} \end{array}$$

MC involves generating a fixed set of normal random numbers $Z∼N(0,I_{N})$ and sampling the random normal process using the “reparameterization trick” [18] where the prediction is sampled as Y=μ(X)+L(X)Z with Cholesky Decomposition of the covariance matrix $\Sigma \left(X,X\right)=L\left(X\right)L{\left(X\right)}^{T}$. Pushing these samples through qEHVI(X) and ∇qEHVI(X) allows us to solve $X^{*}=argmax\;qEHVI\left(X\right)$ using the multi‐start L‐BFGS‐B optimization algorithm. This is an optimizer that uses function evaluations f(x) and gradients ∇f(x) to approximate the Hessian matrix of double derivatives $\nabla^{2}f\left(x\right)$. This approximation speeds up solving $X^{*}=argmax\;f\left(x\right)$ and is commonly used in MOBO and machine learning methods. As we wish to constrain our experiments to achieve some minimum level of growth $y_{min}$ so as not to waste experimental effort in regions of the design space that cannot support cells, we modify qEHVI(X) by multiplying it by an indicator function $\phi \left(x\right)=1\{\mu \left(x\right)\geq y_{min}\}$. Because each point *q* should contribute to the hyper‐volume proportional to the extent to which it satisfies the constraint, we arrive at the multi‐point version of the constrained hyper‐volume function α(X) by averaging out ϕ(x) using the same MC samples. Note that ϕ(x) is not differentiable so we replaced it with a sigmoid function $\phi \left(x\right)\approx \frac{1}{1+exp(-v(x)/\varepsilon )}$ with temperature parameter $\varepsilon =10^{-3}$. We finally arrive at Equation (6) which will be the acquisition function to be optimized throughout this work.

(6) $$\begin{array}{ccc} \alpha (X) & = & \frac{1}{N}\sum_{t=1}^{N}\sum_{X_{j}\in \Omega }\sum_{j=1}^{q}\sum_{k=1}^{K}\prod_{s=1}^{S}{\left(-1\right)}^{j+1}\\ & & \times \;\left[\left({\left[z_{s,k,X_{j},t}\left(x\right)-l_{s,k}\right]}^{+}\right)\prod_{x^{\prime }\in X_{j}}\phi \left(x\right)\right] \end{array}$$

**FIGURE 1:**
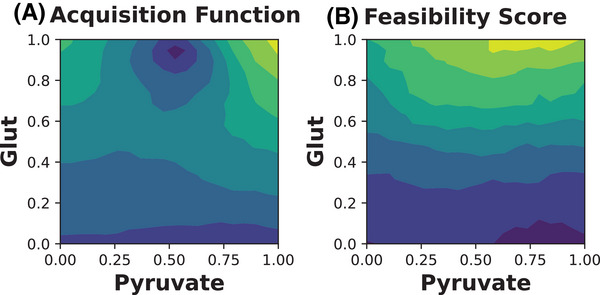
Plot of Acquisition Function and Feasibility Score for q=1 Experiments|(left) expected hyper‐volume improvement α(x) and (right) mean feasibility score ϕ(x) for glutamine and pyruvate concentration (normalized to [0,1]) calculated using N=1000 MC samples. Light/yellow colors represents higher values. For all experiments, the lower bound for predicted growth μ(x) was $l_{\mu }=\bar{\mu }-4\sigma_{\mu }$ (four standard deviations $\sigma_{\mu }$ below the current mean cell growth metric $\bar{\mu }$ across all assays. The lower bound for cost $l_{c}=-1.1$, or 10% above the highest possible value of cost (which, because of our unitless scalarization, is always $c_{max}=1$. Note because α(X) maximizes *S* outputs, we multiply c(x) by ‐1).

An example of α(x) and ϕ(x) is plotted in Figure 1 for glutamine and pyruvate (data collected in this work). The GP model was used to predict the cell growth of the glutamine‐pyruvate design space, which was then used to predict α(x) and the feasibility score ϕ(x). Notice optimizing α(x) may not correspond to maximizing the feasibility of the experiment. This is because cost and uncertainty reduction is considered in α(x) and not ϕ(x).

### 2.6 MOBO algorithm

**FIGURE 2:**
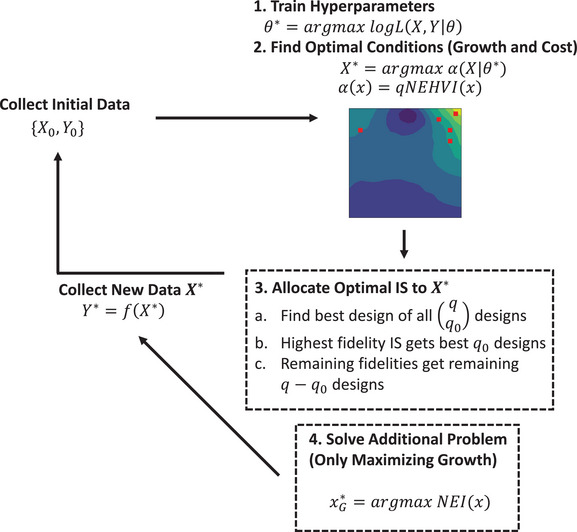
MOBO Algorithm|this loop describes the MOBO algorithm to maximize α(X) that describes the value of a given set of experiments given *q*_0_ high‐fidelity and $q-q_{0}$ low‐fidelity IS samples per batch of experiments. After each batch, the process is repeated until the process is optimized or resources are exhausted. Notes: To increase the presence of high growth conditions, after batch four and nine $y_{min}$ was increased from 0.5 to 0.75 and 1.0, respectively. The minimum standardized variance was thresholded at $\sigma_{min}^{2}=0.02$ but this needed to be changed to 0.05 to reduce numerical stability issues with optimizing α(X). IS, information source; MOBO, multi‐objective Bayesian optimziation.

The MOBO algorithm that designs optimal experiments is shown in Figure 2. After collecting some initial data from a variety of information sources, the model was trained and $X^{*}$ found using multi‐start L‐BFGS‐B for some *q* maximum allowable number of experiments. Because we want to optimize at the high‐fidelity (Passage 2) all calculations in the MOBO algorithm are done using m=0 (see Section 2.4). With $X^{*}$, we now find the optimal fidelity to sample. We started by defining the number of high‐fidelity samples we are willing to measure $q_{0}<q$. α(X) was calculated using Equation (6) for all combinations qq0 in $X^{*}$, and the dominant combination of experiments was allocated to the high‐fidelity metric. The remaining $q-q_{0}$ experiments were allocated to the low‐fidelity set of metrics.

We started our MOBO algorithm in the serum‐free experiments by initialization with 10 Latin Hypercube designs [19]. The algorithm then allocated q=15 experiments with $q_{0}=3$ high‐fidelity and $q-q_{0}=12$ low‐fidelity experiments using the combinatorial heuristic described above. This was repeated for 12 batches of experiments, where a batch is defined as a single group of *q* experiments designed by the MOBO algorithm. Because of the enormous time‐cost of measuring biological replicates of *q*_0_ cell counts for two passages individually, it was assumed that an averaged technical replicate would capture the underlying trends of the system. As the results will show, this did not appreciably detract from the quality optimal media found even over multiple passages. To further bias our experiments towards high growth regions of the design space, after nine batches of experiments we ran an additional high‐fidelity experiment solving $x_{G}^{*}=argmax\;NEI\left(x\right)$ as outlined in [20] where $NEI\left(x\right)=\frac{1}{N}\sum_{t=1}^{N}{\left[max\left\{f_{t}\left(x\right)\right\}-max\left\{f\left(X\right)\right\}\right]}^{+}$. This is equivalent to maximizing the expected improvement of a single experiment of a noisy function without consideration of cost.

### 2.7 Computational environment and packages

Hardware used: Dell Precision 5820 Tower, Intel Xeon W‐2145 DDR4‐2666 Processor (3.7 GHz), 32 GB Memory. Software used: python 3.9.7 (for all programming), gpytorch 1.3.0, pytorch 1.8.1, and botorch 0.4.0 (for modeling and BO), pydoe 0.3.8 (for initialization using Latin Hypercube experiments). For neural network test problem scikit‐learn 0.24.1 was used.

## 3 RESULTS

### 3.1 Computational validation of MOBO method

The MOBO algorithm was tested on the computational test problems introduced in [7] with an additional linear cost function that turned the single‐objective optimization problem into a MOO problem. The results are in Appendixes 3 and 4. α(X) performed as well as previous metrics (a desirability function). Because the hyper‐volume function has fewer controllable design parameters, it was chosen for this novel system. Furthermore, empirical studies of the hyper‐volume [17] and noisy‐hyper‐volume [13] acquisition function indicate that it is superior to a wide variety of MOO and MOBO solvers on synthetic and data‐based optimization problems.

### 3.2 Experimental validation of MOBO method

**FIGURE 3:**
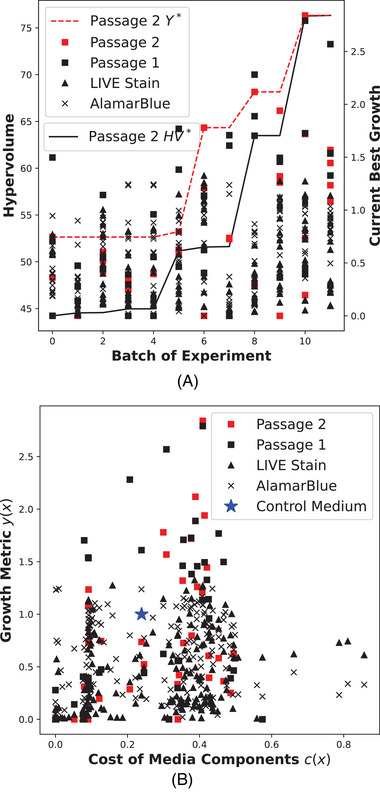
MOBO application to serum‐free system|plot (A) shows improvement in both Passage 2 growth metric and hyper‐volume as the number of designed experiments increases. The dotted line shows the best performing growth experiment per batch and units are on the right‐hand axis. Plot (B) shows the trade‐off between growth and cost from all data and fidelity types.

The most prominent result from the application of the MOBO algorithm to the serum‐free experimental system was the steady improvement in both hyper‐volume and the Passage 2 metric in Figure 3A. Figure 3B shows the trade‐off between cost and growth which is important for designers that need multiple options to consider. Some of the interesting media designs are highlighted in Table 2. Only one medium (CBCB‐0) dominated the control medium in both growth and cost, resulting in 23% more growth at 62.5% of the cost of the control. CBCB‐0 had notably lower concentrations of major growth factors like insulin, transferrin, FGF2, and TGFβ1 and higher concentrations of progesterone, estradiol, IL6, and LIF. CBCB‐1 was another interesting medium that had 78% more growth at only 25% additional cost compared to the control. This was due to higher concentrations of the growth factors that CBCB‐0 lacked. Finally, CBCB‐2 and CBCB‐3 had a 112% and 184% improvement in growth at an increase in cost of 62% and 71% over control respectively. CBCB‐2 and CBCB‐3 had even higher concentrations of both the insulin, transferrin, FGF2, and TGFβ1 growth factors, while also elevating the concentration of all factors from progesterone to Pigment epithelium‐derived factor (PEDF).

**TABLE 2:** Optimal Media|groups of media that lie on or near the trade‐off curve. Only CBCB‐2 was found by maximizing NEI(x) rather than α(X). The concentration (mg/mL) of all media was between the minimum and maximum listed in Table 1. The cost is a unitless metric of relative economic cost of each component or group.

	**CBCB‐0**	**CBCB‐1**	**CBCB‐2**	**CBCB‐3**	**Control**
**NEAA** ^a^	0.75x	1.50x	1.55x	1.20x	1.00x
**EAA** ^a^	0.90x	1.35x	1.40x	0.95x	1.00x
**V** ^a^	3.60x	4.30x	2.25x	1.60x	1.00x
**Salt** ^a^	0.50x	2.50x	1.75x	0.90x	1.00x
**Metals** ^a^	5.00x	4.80x	3.40x	3.55x	1.00x
**DNA** ^a^	1.95x	3.05x	2.95x	2.00x	1.00x
**Fat** ^a^	2.75x	1.25x	2.20x	2.65x	1.00x
**SS**	4.41E‐05	4.83E‐05	3.99E‐05	4.41E‐05	1.40E‐05
**AA**	0.18	0.21	0.23	0.23	0.06
**Gluc**	1.62	9.59	3.51	6.89	4.05
**Glut**	1.35	1.95	1.58	1.54	0.43
**Pyruvate**	0.20	0.25	0.13	0.11	0.06
**NaCl**	4.76	4.76	5.60	3.64	7.00
**I**	0.01	0.01	0.02	0.02	0.10
**T**	5.00E‐03	3.35E‐02	1.95E‐02	2.20E‐02	1.00E‐02
**FGF2**	3.00E‐05	1.29E‐04	1.32E‐04	1.35E‐04	9.00E‐05
**TGFb1**	1.00E‐06	1.00E‐06	1.70E‐06	2.80E‐06	2.00E‐06
**EGF**	4.25E‐06	2.25E‐05	9.25E‐06	1.10E‐05	0.00
**P**	1.73E‐05	5.25E‐06	1.93E‐05	1.50E‐05	0.00
**Estra**	5.75E‐06	5.00E‐07	4.75E‐06	2.38E‐06	0.00
**IL6**	1.13E‐05	5.00E‐06	3.94E‐05	3.94E‐05	0.00
**LIF**	3.75E‐07	8.75E‐07	4.00E‐06	1.63E‐06	0.00
**TGFb3**	0.00	0.00	4.48E‐06	7.36E‐06	0.00
**HGF**	0.00	0.00	1.00E‐06	2.00E‐06	0.00
**PDGF**	0.00	0.00	9.25E‐06	9.50E‐06	0.00
**PEDF**	0.00	0.00	2.50E‐06	3.75E‐06	0.00
**Growth**	1.23	1.78	2.12	2.84	1.00
**Cost**	0.09	0.30	0.39	0.41	0.24

^a^ The max/min concentration is relative to stock concentrations in Appendix 1. All media have a sodium bicarbonate concentration of 2.44 mg/mL and were stored at 5°C.

### 3.3 Long‐term proliferation

**FIGURE 4:**
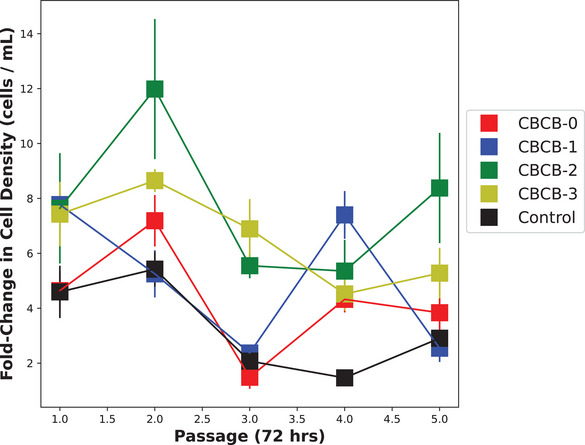
Long‐term proliferation|C2C12 were grown for an additional five passages. Standard deviation of cell density shown as shaded region indicating experimental error in Countess II automatic cell counter. Fold‐change in cell density refers to the ratio of 72 h cell density to seeding density (60,000 cells/well).

We then tested all the highlighted media for five passages to assess the ability of our Passage 2 high‐fidelity metric to mimic longer‐term effects of the media on growth (the effect of passaging and attachment that are difficult or impossible to evaluate with the low‐fidelity data sources). Figure 4 shows the fold‐increase in cells (counted using the automatic cell counter) over the initial 60,000 cells seeded over five sequential passages. CBCB‐2 performed the most robustly over time but all designed media performed as well as or better than the control. We also compared our control to commercial E8 and found no significant differences in growth over five passages (data not shown).

### 3.4 Sensitivity analysis

**FIGURE 5:**
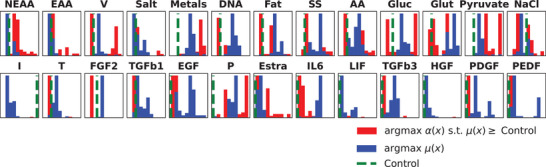
Distribution of optimized samples for sensitivity analysis|distribution of optimal concentrations (x‐axis) and count (y‐axis). Samples were taken from 50 randomized restarts of L‐BFGS‐B optimization algorithm under two conditions: The max growth condition solves argmaxμ(x) to show what concentrations result in high growth without considering cost (dark, blue). The max‐α(x) analysis solves argmaxα(x)s.t.μ(x)≥Control which attempts to consider cost while achieving higher growth than the control (light, red).

We now use sensitivity analysis to understand what high‐fidelity behavior is being captured given the data. One way of doing this is to run Step 2 of the MOBO algorithm described in Figure 2 to find a single q=1 optimal medium combination at random starting locations (optimizers like L‐BFGS‐B require a starting point) and observing the results from several runs. First, we maximized α(x) under the constraint that μ(x)≥Control in order to observe what sets of media combinations are predicted to produce more growth than the control while penalizing high cost. Next, we maximized only μ(x) in order to observe what media might maximize growth regardless of cost. The two distributions are plotted in Figure 5. The max growth condition had generally higher concentrations of most growth factors but not all basal components. This confirms the previous section where higher growth was achieved through higher growth factor concentrations, particularly transferrin, FGF2, TGFβ1, EGF, TGFβ3, and PDGF.

**FIGURE 6:**
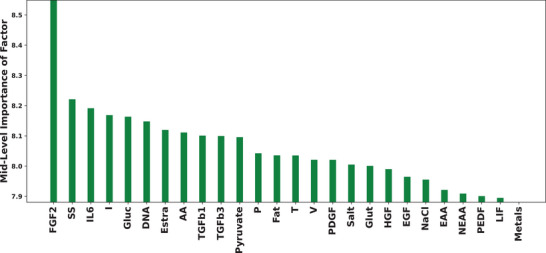
Mid‐level VARS variogram|ordered by highest to lowest for the h=0.3 integrated variogram. The height of the bar indicates the relative importance of that component between randomized synthetic media sampled at most with 30% Euclidean distance‐differences in concentration. The plot was generated using 1000 random samples in the design space (whereas the methods described in [21, 22] utilize the STAR method).

Figure 5 only tells us what the model thinks are the best conditions and not the relative magnitude of each factor on growth. As a further means of quantifying this, we computed an “integrated variogram”‐metric using the VARS technique described in [21, 22] (Figure 6) for each component. This VARS sensitivity analysis suggests that FGF2, IL6, TGFβ1, and several basal components had significant effects on growth. This mostly confirms the previous section that FGF2, TGFβ1, and several other growth factors had a large effect on growth, but it is impossible to say anything more suggestive than that.

## 4 DISCUSSION

The MOBO algorithm was successful because a robust, long‐term data set was built over time, improving the model as more data was collected. Additionally, α(X) was tailored to generate high‐value experiments near the trade‐off curve between cell growth and media cost. A separate constraint function ϕ(X) translated our need to primarily search for high‐growth designs into a mathematical function, as we expected most of the design space to not support cell growth. Some shortcomings of this work are that (i) we did not compare our MOBO method to an equivalent design of experiments (DOE) method, though we have previously shown similar methods are significantly more efficient than traditional DOEs such as response surface methods [5, 7]. Additionally, (ii) Figure 4 indicates media performance tended to decrease over time. This could be due to morphological changes, physical damage due to passaging, or accumulation of toxins and waste products. Clearly, our Passage 2 metric was not enough to fully predict the changing dynamics of cell growth over greater than two passages, though it did so reasonably well given the significant savings in experimental time and resources. (iii) Separate experiments with bovine satellite cells (data not shown) also indicated that none of the highest performing media supported growth over multiple passages, thus limiting the generalizability of our media to only our C2C12 cells adapted to commercial Essential 8 without Matrigel. However, such a result does indicate the need to re‐optimize media and environmental conditions when studying new cell types or cells with significant genetic or metabolic changes, as such our methods could prove even more useful. This is particularly the case in industrial biotechnology applications such as cellular agriculture where new products and processes are being introduced at a rapid pace. Finally, (iv) C2C12 cells are much easier to culture over multiple passages compared to cells that might be used in cellular agriculture, such as animal satellite cells, therefore our exact methodology for preparing the cell banks (such as the preconditioning portion of the study) would have to be customized for less robust cell lines. Attachment factors or Matrigel might be required to allow the cells to proliferate. Different cell lines also have different metabolic needs which might be stimulated with different signaling pathways, and thus require growth factors not described in this study. Industrial applications would also require many more cells than what is generated from two passages, militating for a higher “high‐fidelity” assay that might preclude our MOBO method. These facts underscores the necessity to pair this kind of black‐box approach with more traditional “white‐box” where cellular processes that cannot be easily modeled for learned using BO methods are understood using traditional biological techniques (such as cell isolation, cloning, and omics analysis).

In general, the MOBO algorithm was able to design media according to the objective function we picked for this system. We were able to identify several media with >100% more growth relative to the control, as well as a medium with 23% more growth at only 62.5% of the cost of the control (CBCB‐0). This allows for further, more principled, experiments to be made to accompany and expand on the discoveries made in this study such as spent‐media analysis and morphological studies of the cells after exposure to our media. The MOBO algorithm was also able to discover novel media, such as CBCB‐2, well‐suited to long‐term C2C12 growth far beyond the limits of this study. This would make it an interesting candidate for large scale production such as in cellular agriculture. Further work should be performed on correlating biomarkers and morphological attributes to cell differentiation and proliferation, both to improve the robustness of predictions and to simultaneously optimize proliferation and differentiation. Even without these improvements, this work is still relevant to those interested in quickly optimizing their media formulations, generally in the serum‐free case, and particularly in the case of difficult‐to‐measure objectives such as long‐term cell growth.

## 5 PRACTICAL APPLICATION

The field of cellular agriculture, whereby animal products are grown in bioreactors for food production, has the potentially to reduce the negative environmental and ethical externalities of meat production. In this work, we design a serum‐free medium for such applications using an active learning BO method for the low‐cost proliferation of C2C12 mammalian muscle cells. The algorithm automatically discovers optimal combinations of media components by allocating a battery of growth assays within a closed learning loop. Through this process, several promising novel media designs were discovered which demonstrated robust cell growth at low cost.

## CONFLICT OF INTEREST STATEMENT

We have no conflicts of interest to disclose.

## Data Availability

The data that support the findings of this study are openly available in at https://github.com/ZacharyCosenza/GradStuff_Cosenza.
